# The Cinderella of positive psychology: spiritual well-being as an emerging dimension of flourishing in pastoral work

**DOI:** 10.1080/17482631.2023.2170767

**Published:** 2023-02-13

**Authors:** Elizabeth Cornelia Rudolph, Antoni Barnard

**Affiliations:** a Department of Human Resource Management, College of Economic and Management Sciences, Muckleneuk Campus, University of South Africa, Pretoria, South Africa; b Department of Industrial and Organisational Psychology, College of Economic and Management Sciences, University of South Africa, Pretoria, South Africa

**Keywords:** Pastors, well-being, spiritual well-being, flourishing, interactive qualitative analysis, IQA, narrative synthesis, interpretive pragmatic paradigm

## Abstract

**Purpose:**

The invaluable, yet challenging role of pastors in the community signifies the need to understand and care for their well-being. Well-being, conceptualized in the multidimensional construct of flourishing, does not explicitly include spiritual well-being, yet, it is the foundation of pastors’ well-being. In this article we aim to describe pastors’ spiritual well-being and in so doing, highlight its fundamental importance in pastors’ flourishing in the ministry.

**Methods:**

Positioned in the interpretive pragmatic paradigm, data were gathered and analysed from three focus groups with 18 pastors in the Dutch Reformed Church and the Uniting Reformed Church of South Africa. Interactive qualitative analysis was applied, and results were conceptually refined through narrative synthesis.

**Results:**

Four themes were constructed to describe pastors’ spiritual well-being namely: i) an altruistic calling; ii) discipleship iii) seasons of the ministry; and iv) ethics.

**Conclusion:**

The findings highlight the importance and essence of the spiritual aspects predominant to pastors’ well-being. Attending to spiritual well-being will enhance their resilience and constructive coping and is integral to their way of flourishing at work. This proposes an extension of the flourishing framework to include spiritual well-being as an explicitly conceptualized sub-dimension for application to the study’s Christian pastoral context.

## Introduction

Spiritual well-being has an important function in life because it serves as a socio-psychological resource for coping with challenging life experiences (Bangcola & Pangandaman, 2022; Charzyńska, 2015). The importance of spirituality in the workplace for employee well-being is widely acknowledged (Bester & Müller, 2017; Gotsis & Grimani, 2017) and revered for contributing to employees’ positive coping and resilience (Bangcola & Pangandaman, 2022). It has been ascribed to several desirable processes and outcomes, such as quality relationships, employees with purpose and meaning, optimal organizational functioning and sustainability, and effective transformation and change (Fry & Egel, 2017; Nzonzo, 2017). In terms of coping and well-being, spiritual resources have the most significant impact on professionals in the ministry (Darling et al., 2004). Specifically, spiritual well-being lies at the core of pastors’ overall well-being (Rudolph, 2019) and remains an imperative topic for well-being research in the pastoral context (Kansiewicz et al., 2022; Louw, 2018).

Professionals within the Christian ministry, globally and in South Africa, experience unique work pressures characteristic to the role they fulfil in society (Buys & Rothmann, 2010; Chan & Chen, 2019; Pooler, 2011). Because society holds idealistic expectations of the pastor, pastoral work easily becomes all-consuming, taxing the pastor’s well-being and placing them at risk for loneliness, burnout, distress and even moral misconduct (Pooler, 2011). Pastoral work has been described as high-demand, low-reward (Chan & Chen, 2019); prone to role overload, role conflict and burnout (Rosendahl & Rosendahl, 2020); filled with personal boundary ambiguity (Chan & Chen, 2019); and replete with emotional labour (Adams et al., 2017). Some have noted that pastors’ high burnout levels and ill-being are unavoidable (Grant & Niemandt, 2015; Muse, 2007) and higher than the average in most professions (Hessel, 2015). This unique psychological and spiritual burden (Rudolph & Landman, 2019) signifies caring for pastors’ spiritual well-being as a vital endeavour to support these helping professionals cope and thrive in their work (Louw, 2017, 2018). Pastors’ resilience is attributed to spiritual well-being, emphasizing the need to study and cultivate their spiritual well-being in a context specific manner (Adams et al., 2017; Meeden, 2022). Spiritual well-being is moreover dynamic in nature and more research generating conceptual understanding is required (Bangcola & Pangandaman, 2022; Ghaderi et al., 2018).

This article stems from a broader study on coaching pastors for sustained well-being. The aim of this paper is to describe pastors’ spiritual well-being, which emerged from the broader study’s findings, as fundamental to pastors’ overall well-being (Rudolph, 2019). Whilst the emotional, psychological, and social dimensions of flourishing were evident in the pastors’ well-being experiences, phenomena relevant to conceptualizing spiritual well-being were palpable in the data. Caring for the pastor’s well-being is of utmost importance to maintain resilient clergy and healthy congregations that positively impact society (Adams et al., 2017; Pooler, 2011). Such caring can best be done if what constitutes the pastor’s well-being is understood. Before presenting the findings, spiritual well-being and well-being as encapsulated in the multidimensional concept of flourishing are presented, followed by a discussion of the research methods. Lastly, the findings are discussed, with implications and recommendations.

### Spiritual well-being

Spiritual well-being is a fundamental human need which, if fulfilled, leaves one with a sense of purpose and meaning and belonging (Egel & Fry, 2017; Hunsaker, 2020). Calling and membership are therefore the primary conceptual pillars of spiritual well-being (Gotsis & Grimani, 2017). Calling refers to engaging in activities that give the individual a sense of purpose and meaning and the experience of making a positive difference in the lives of others (Yang & Fry, 2018). Membership is reflected in the sense of belonging and community and feeling understood and appreciated, leading to positive organizational and extra-role behaviours (Hunsaker, 2020) or to the care and concern for others as reflected in altruistic love (Egel & Fry, 2017). The expression of altruistic love as an element of spiritual leadership and well-being is consequent to a sense of interconnectedness, a person’s capacity to connect with something beyond oneself (Egel & Fry, 2017; Gotsis & Grimani, 2017). The degree to connect with something beyond or greater than the self is also referred to as a sense of transcendence and relates to existential relationships with the self, others, nature and/or a higher being (Gomez & Fisher, 2005). This relational perspective on spiritual well-being is encapsulated in the well-known spiritual health framework proposed by J. Fisher (2010). According to J. Fisher’s (2010) perspective, spiritual well-being is a dynamic state that reflects the extent to which people experience harmony in their relationships with themselves (personal), with other people (communal), with the environment (environmental), and with God or a transcendental other (transcendental) (Ghaderi et al., 2018; Gomez & Fisher, 2005; J. W. Fisher et al., 2000). Spiritual well-being has also been conceptualized along two distinctive but interrelated dimensions: existential and religious well-being (Malinakova et al., 2019; Ziapour et al., 2017). The horizontal (existential) dimension refers to life purpose and satisfaction (Malinakova et al., 2019), self-integration and reliance on inner resources (Alorani & Alradaydeh, 2018). The vertical (religious) dimension refers to one’s sense of connection with God or a higher power or being (Alorani & Alradaydeh, 2018; Ziapour et al., 2017).

### Flourishing

In the field of positive psychology, flourishing is an all-encompassing well-being construct and flourishing research increases knowledge on how people achieve optimal functioning at work (C. L. M. Keyes, 2007; Janse van Rensburg et al., 2017; Rothmann, 2013; Seligman, 2011). Conceptualisations of flourishing entail characteristics of well-being such as eudaimonia, hedonia, subjective well-being (SBW), and psychological well-being (PSW) (C. L. Keyes & Annas, 2009; C. L. M. Keyes, 2002, 2005, 2007; Seligman, 2011). Originally conceptualizations of flourishing at work focussed on intrapersonal functioning (C. L. Keyes & Annas, 2009) but was expanded to include interpersonal functioning (Seligman, 2011), highlighting the following elements to determine whether people thrive in life: positive emotion, engagement/interest, self-esteem, optimism, resilience, and positive relationships. These well-being indicators of flourishing are categorically summarized as feeling good and functioning well (C. L. M. Keyes, 2009; Nzonzo, 2017) but reflects a multidimensional state of emotional, psychological and social well-being (Janse van Rensburg et al., 2017; Rothmann, 2013). The growing body of knowledge about employee well-being has therefore come to establish flourishing as a multidimensional well-being framework (Bester & Müller, 2017; Seligman, 2011). The work of Rothmann (2013) is seminal in this regard as it provides a conceptual framework to integrate the multidimensionality of flourishing in a useful way. Rothmann (2013) conceptualizes flourishing according to three dimensions, namely emotional well-being, psychological well-being, and social well-being. In the work context, emotional well-being manifests as job satisfaction and positive affect. Psychological well-being manifests as self-determination, engagement, purpose and meaning, and harmony. Social well-being is evident in social acceptance, social growth, social contribution, and social coherence. Rothmann’s (2013) conceptualization of flourishing reflects the work of C. L. M. Keyes (2002, 2005) and Seligman (2011), and his framework excludes spiritual well-being as an explicit dimension. As such we metaphorically pose spiritual well-being as the Cinderella^1^ of positive psychology and the flourishing framework.

## Methods

### Study design

The study followed a multimethod qualitative inquiry based on an interpretive-pragmatist stance (Goldkuhl, 2012), applying both interactive qualitative analysis (IQA) (Northcutt & McCoy, 2004) and narrative synthesis (Lisy & Porritt, 2016; Popay et al., 2006) Congruent to an interpretive-pragmatist paradigm, IQA is a qualitative methodology that presupposes a social constructionist epistemology and provides a systemic, rigorous, and credible framework of inquiry (Cook & Geldenhuys, 2018). IQA was chosen because of its systematic rigour and because participants have an active role in co-constructing knowledge with the researcher (Bargate, 2014; Tabane & Human-Vogel, 2010). Narrative synthesis is also an interpretive methodology that focuses on exploring similarities, differences, and relationships in data to yield a robust conceptual understanding rooted in the strength of comparative evidence across data (Arya et al., 2017; Lisy & Porritt, 2016).

The IQA design entails concurrent data gathering and analysis through focus groups (Bargate, 2014). Applying the IQA focus group protocol, the researcher facilitates a systematic group process that empowers participants to generate and analyse data in line with the research objective (Northcutt & McCoy, 2004; Tabane & Human-Vogel, 2010). The three focus groups in this study generated several themes (known as *affinities* in IQA terminology) and grouped the affinities to produce a Systems Influence Diagram (SID) which is a conceptual framework describing the research phenomenon (Northcutt & McCoy, 2004). Because of the nature of IQA, a transparent audit trail is evidence of how the individual participants interactively determined the themes and the resulting SID in the structured focus group sessions (Albertyn et al., 2018; Bargate, 2014). A narrative synthesis was then applied to synthesize meaning across the focus groups’ themes and their resulting three SIDs. The narrative synthesis intended to elevate the depth of affinity conceptualization, enhance analytic rigour, and produce a robust integrative understanding of the research phenomenon (pastors’ well-being). Whilst the IQA protocol focuses on allowing participants to generate, analyse and interpret their own data (Cook & Geldenhuys, 2018), the narrative synthesis enabled the researchers to further construct knowledge through comparative analysis across three focus group findings whilst iteratively considering their preconceptions rooted in well-being theory (flourishing and spiritual well-being) (see Arya et al., 2017).

### Setting

The research was set in a South African-based, unified Presbyterian synodical church structure. This church is fundamentally Christian and includes two denominations, namely the Dutch Reformed Church (DRC) and the United Reformed Church of South Africa (URCSA) (Kuyler, 2015), which constituted the two main constituencies of this study. According to Statistics South Africa reports, the religious affiliation in South Africa is predominantly Christian (85.6%), followed by Muslim (2%), ancestral, tribal, animist or other traditional African religions (5%), Hindu (1%), Jewish (0.2%), other religions (0.3%), no religion (5.6%) and people who do not know (0.3%) (Stets & Serpe, 2013, p. 32). A constituency refers to a group of people regarded as an authority on the research phenomenon and has a shared understanding of the phenomenon (Northcutt & McCoy, 2004). Participants for an IQA study are chosen as representatives of a constituency (Cook & Geldenhuys, 2018).

### Sampling and participants

The sampling process underlying IQA is based on the principle of commonality, which is akin to setting inclusion criteria in purposeful sampling. The principle of commonality requires participants in a focus group to share common experiences, work or live within some common structure and/or have a similar background (Albertyn et al., 2018; Northcutt & McCoy, 2004). Other IQA guidelines for inclusion require participants to be information rich regarding the research phenomenon; have the ability to reflect; and be available and willing to participate (Northcutt & McCoy, 2004). In accordance with the principle of commonality, participants were included if they had a professional theology qualification and were ordained pastors in the DRC or the URCSA. Their professionality also alluded to their ability to reflect. To ensure richness of experience, participants were included if they had a minimum of one year’s pastoral experience in a congregation. Availability was the only inclusion criterion that distinguished some pastors to be excluded because they were not available when the focus groups were scheduled.

A total of eighteen pastors agreed to participate and were allocated to focus groups as representatives of either a DRC or a URCSA constituency. To account for a workable focus group size (see Onwuegbuzie et al., 2009; Suri, 2011), two DRC focus groups and one URCSA focus group were conducted. Focus group 1 (a DRC constituency) consisted of eight participants (*n* = 8), focus group 2 (a DRC constituency) had six participants (*n* = 6) and focus group 3 (an URCSA constituency) had four participants (*n* = 4). Participants’ age varied between 31 and 65 years. All the participants held a qualification in Theology and their years of experience in the ministry varied between 11 and 36 years. Only one participant had less than five years’ experience and worked under supervision as a pastoral youth worker for a congregation. In Table 1 the demographic information of participants per focus group is illustrated. For confidentiality purposes pseudonyms are used for participants to protect their identity and personal information.

**Table I.** Demographic information of participants per focus group.

Participants (Pastors)	Age (Years per category)	Education (Degree)	Ministry experience (Years per category)	Years in current congregation (years per category)
FOCUS GROUP 1 (FG1)				
DRC GROUP; *n* = 8				
Tessa	36 – 40	Master’s	11 – 20	6 – 10
*Bernard	61 – 65	Honours	*36+	*31+
Jaco	61 – 65	Master’s	26 – 30	31+
Hennie	41 – 45	Master’s	11 – 20	11 – 20
Arrie	56 – 60	Honours	31 – 35	11 – 20
Diederick	41 – 45	Master’s	11 – 20	6 – 10
**Francois	31 – 35	Honours	**1 – 5	**1 – 5
Nico	56 – 60	Honours	26 – 30	26 – 30
FOCUS GROUP 2 (FG2)				
DRC GROUP; *n* = 6				
Werner	36 – 40	Master’s	11 – 20	1 – 5
Neels	56 – 60	Master’s	26 – 30	21 – 25
Peet	51 – 55	Doctorate	26 – 30	11 – 20
Drikus	46 – 50	Doctorate	21 – 25	21 – 25
Henro	51 – 55	Master’s	21 – 25	11 – 20
Isak	41 – 45	Honours	11 – 20	6 – 10
FOCUS GROUP 1 (FG1)				
DRC GROUP; *n* = 8				
***Petrus	61 – 65	Undergraduate	21 – 25	6 – 10
Johannes	56 – 60	Honours	11 – 20	6 – 10
Moses	61 – 65	Honours	11 – 20	6 – 10
Mathew	51 – 55	Honours	11 – 20	11 – 20

*Rev. Bernard was in the retirement phase of his career at the time of data collection and analysis.

**Rev. Francois is relatively new to the ministry but works under supervision as a pastoral youth worker in his current congregation.

***Rev. Petrus is a registered pastor at the URCSA.

Source: Rudolph (2019, p. 155).

### Data collection

**Figure 1.**
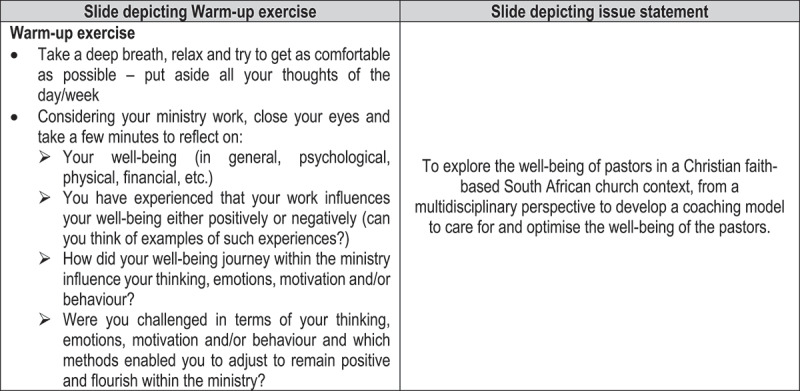
Warm-up exercise and silent brainstorming (Individually) slides.

Source: Rudolph (2019, p. 130–133)

Data were collected during three focus group sessions, conducted following IQA protocols (Northcutt & McCoy, 2004) in June 2014. An IQA focus group commences with a warm-up exercise to establish rapport and focus participants’ attention on the broader research topic and context. After the warm-up exercise, the same issue statement was used by the facilitator^2^ for all three focus groups to further focus them for the brainstorming sessions. Figure 1 depicts the content of the slide used for the warm-up exercise and of the slide showing the issue statement. The issue statement remained projected and visible to all throughout the session. The facilitator continued to systematically facilitate silent and collaborative brainstorming exercises. These exercises enabled participants to share their experiences, thoughts, and emotions about the issue statement. Silent brainstorming is an individual exercise in which each participant notes their thoughts, feelings, and reflections concerning the issue statement on notecards without any communication. They then post notecards randomly on a whiteboard for all to see (see Northcutt & McCoy, 2004).

The facilitator then systematically focussed on each notecard and allowed participants to share personal stories about how the note reflected or demonstrated their well-being in the ministry. This was followed by an interactive dialogue between participants resulting in the development and categorizing of affinities and sub-affinities. Affinities refer to the themes that describe or explain the research phenomenon. The focus group sessions were audio-recorded with participants’ consent and transcribed by the facilitator.

### Data analysis

With data collection and analysis being concurrent processes in IQA methodology, two analytic strategies consequent to affinity production (described above) are isolated by Northcutt and McCoy (2004), namely, theoretical coding and the construction of a SID. Both strategies form part of the focus group session, and participants in effect conduct the data analysis according to the IQA analytic protocol.

**Figure 2.**
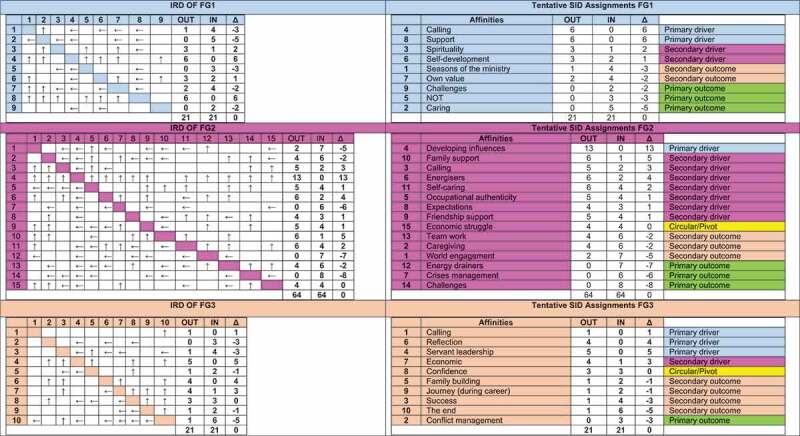
Focus Groups’ IRDs and Tentative SIDs.

Source: Rudolph (2019, p. 171)

During theoretical coding, the construction of an Affinity Relationship Table (ART) and an interrelated diagram (IRD) is facilitated through a voting protocol on the relational nature between affinity pairs (Northcutt & McCoy, 2004). Affinities are first listed in pairs, and IF/THEN statements are used to describe the relationships within each affinity pair (eg. IF [affinity XYZ] THEN [affinity ABC]). These relationship statements are summarized in an ART, noting the IF/THEN direction of each affinity relationship pair. Participants secondly vote on the importance of the relationship between all affinities. The cumulative frequency of votes is calculated for each affinity pair. If an affinity has a causal effect (IF/THEN) on another affinity, it is illustrated with an IN. If not, it is illustrated with an OUT. The cumulative INs and OUTs for each affinity is then indicated in the IRD (see Figure 2). Delta (Δ) indicates the strength of each affinity in the cause and effect affinity system. This process determines the affinities that best describe pastors’ well-being and classifies the functions of the affinity as being either a driver, circular/pivot, or outcome in conceptualizing pastors’ well-being. Ascribing functions to affinities result in a SID and reflects a summative visual mind-map of the results (Bargate, 2014; Northcutt & McCoy, 2004). The three resulting SIDs in this study are also depicted in Figure 2 – each SID is depicted alongside its IRD origin.

As IQA does not provide a consequent analytic for comparative synthesis across distinct focus group data sets, we applied narrative synthesis. The narrative synthesis enabled collating the three SIDs into one by considering affinities that either had the same or different meanings and functions in the three focus group systems (Northcutt & McCoy, 2004; Onwuegbuzie et al., 2009). Being mindful of the similarities in meaning ascribed by participants to affinities and the meta-theoretical foundation of the study, affinities were conceptually refined as themes describing the pastors’ well-being.

### Ethics and trustworthiness

Ethical clearance for this study was obtained from the relevant institutional college ethics committee in compliance with the University of South Africa (UNISA) policy on research ethics (Ref#: 2014/CEMS/IOP/001). Permission to conduct the study was granted by the Presbyterian Synod of the Dutch Reformed Church and the Uniting Reformed Church of South Africa, and the primary researcher was given access to potential participants’ contact details. Participants were contacted via telephone and email. After initial telephonic contact, the details of the study were communicated via email. This entailed the purpose and nature of the research and participants’ roles and rights regarding freedom to withdraw, anonymity, and confidentiality. All participants provided written consent for their participation and data recordings and dissemination of findings based on the data for research purposes. To ensure the confidentiality of participants’ personal information, pseudonyms were used, and all necessary precautions were taken to minimize any unique identifiers of those who participated in the study.

To enhance the trustworthiness of the study, several strategies were employed. To ensure skilled application of the IQA design, the first author attended an IQA certification workshop by Dr Northcutt (one of the originators of the IQA method) at Stellenbosch University. Both authors attended another IQA methodology workshop presented by Dr Albertyn, a published IQA expert (see Albertyn et al., 2018). While the structured IQA process provides a platform for participants to generate and structure data, the IQA focus group protocol also offers participants several opportunities to confirm and reconfirm the data generated and the meanings ascribed to the data. Moreover, the primary researcher consistently reviewed focus group results with the second researcher, which lead to the consequent refinement of the data through narrative synthesis. Data and findings were also corroborated by various experts. Regular reviews with an IQA expert ensured the correct application of IQA analytic protocols. A registered psychologist was consulted to account for integrating the data with flourishing theory. Lastly, the findings were also reviewed with a theology professor at the University of South Africa to reflect on theological interpretations of the data.

## Results

The results are discussed by first reporting on the findings from the IQA process. This includes a report on the results of the affinity production, the theoretical coding and the final SID. Second, the findings of the narrative synthesis are presented in four themes that were constructed from the IQA findings to denote the conceptual meaning of pastors’ spiritual well-being. While other themes were also constructed as descriptive of pastors’ overall well-being in the broader study, these fall out of the scope of this paper.

### Affinity production

The affinity production phase across the three focus groups actively generated 184 notecards with 34 affinities. The total is broken down in Table 2, depicting the total number of notecards, sub-affinities and affinities per focus group (FG). Sub-affinities are reduced by removing duplicated notecards and through a collaborative decision on the importance and relevance of notecards.

**Table II.** Total number of notecards, sub-affinities, and affinities**.

	FG*1	FG2	FG3	Total
Number of notecards	67	81	36	184
Number of sub-affinities	63	74	35	172
Number of affinities	9	15	10	34

* FG = Focus group.

**The word “affinities” means themes, as explained earlier in the article.

Source: Rudolph (2019, p. 158).

### Theoretical coding

An ART was constructed following the voting protocol on affinity pair relationships via IF/THEN statements. The ART illustrates the nature of the affinity pairs (i.e., the affinity pair direction of influence) and the frequency of votes cast for each affinity pair. FG1 cast a total of 214 votes for 36 possible relationships between affinity pairs, FG2 cast 575 votes for 105 potential relationships, and FG3 cast 148 votes for 45 possible relationships. Votes were calculated according to cumulative frequency for each affinity and summarized in an IRD (left tables in Figure 2 below). The IRD comprises a matrix depicting all the hypothesized cause-and-effect affinity relationships indicating which affinity directly influences the other in an affinity pair or whether there is no relationship in the affinity pair. The frequencies from the IRDs are used to derive a tentative SID, which is evident in the tables to the right in Figure 2.

### Systems influence diagram

From the tentative SIDs, uncluttered SIDs are constructed by re-ordering the affinities, with the IN/OUT relationships in descending order of delta and following an uncluttering process based on the original ART. From the ART, IF/THEN statements guide removing redundant links between affinity pairs and finding alternative routes/paths from one affinity to another in the system (Northcutt & McCoy, 2004). The final three SIDs represented the primary and secondary drivers (causes), pivots/circular, and primary and secondary outcomes (effects) within the system for each focus group. A primary driver indicates a significant cause because it affects many other affinities but is not affected by others. A relative cause or influence on affinities in the system is represented as a secondary driver. Circulars or pivots are affinities for which there are equal numbers of influences by and on other affinities. A primary outcome indicates an affinity with a significant effect because many affinities cause it, but others do not. Lastly, an affinity identified as a secondary outcome reveals a relative impact or influence on other affinities in the system (Northcutt & McCoy, 2004). The resulting SIDs for the three focus groups are summarized in Table 3 below, indicating a total of 34 affinities. According to their functional roles, the clustering of the affinities in this table constituted the start of the narrative synthesis.

**Table III.** SID per focus group: drivers, pivots, and outcomes.

	FG*1	FG2	FG3
Primary Drivers	Support	Developmental influences	Servant leadership
	Calling		Calling
			Reflection
Secondary Drivers		Self-caring	
		Teamwork	
Pivots/Circulars	Seasons of the ministry	Calling	Success
	Self-development	Family support	Family building
		Friends support	Confidence
		Economic Struggle	Economic
		Occupational authenticity	Career journey
		Energisers	
		Expectations	
		Energy drainers	
		World engagement	
Primary Outcomes	Challenges	Challenges	Conflict management
	Caring	Crises management	
	NOT		
Secondary Outcomes	Own value	Caregiving	The end

* FG = Focus group

Source: Rudolph (2019)

### Narrative synthesis

The narrative synthesis entailed a qualitative comparison across the respective focus groups’ drivers, pivots, and outcomes. It resulted in thematically reducing 34 affinities into eight primary themes describing pastors’ well-being. Reducing thematically entailed synthesizing affinities with similar functional labels and similar conceptual meanings. Some affinities were similarly labelled across focus groups and clearly related to the meaning ascribed to them by participants (e.g., Caregiving, Caring, and Servant leadership). The original qualitative data reflecting participants’ narrated meaning of the affinities in the IQA focus groups were revisited and critically reflected on for conceptual relatedness.

Further synthesis and meaning construction developed from comparing focus groups’ affinity conceptualizations with relevant literature on spiritual well-being. In view of the objective of this article, four themes were taken from the eight themes describing pastors’ well-being, as most poignant in describing pastors’ spiritual well-being. The four themes were thus conceptually derived from the comparative analysis of the IQA findings, rooted in the verbatim data that were generated in the IQA focus groups and interpreted against literature on spiritual well-being. The four themes include *calling*, *discipleship, seasons of the ministry*, and *ethics*.

#### An altruistic calling

Table 3 shows that the affinity referred to as *calling* was perceived similar across all three focus groups (as a primary driver for FG1 and FG3 and as a pivot, FG2), and consequent to the narrative synthesis, it was adopted as a theme describing a primary driver in pastors’ spiritual well-being.

Spiritual well-being was specifically related to calling and described by FG1 as a conviction and a belief that God called them to become servants of God. Jaco (FG1), for example, directly relates spiritual well-being to the internalized sense of being called to serve God: “*I think the importance for pastors to remain well is a conviction, God called me … [it is] an emotional, spiritual experience that I felt obligated to*”. Other participants note how their calling originated from elders or respected role models, such as Moses (FG3) recalling a doctor telling his mother: “*this young boy, the way he behaves, his personality, I think the right profession is to become a pastor*”. He continues: “*It started there in my mind … I thought about [the words of the doctor] and took this decision”*. For FG3 participants, calling originated from significant life experiences, leading to a relationship with God and the pastoral career choice. The story of Johannes’ (FG3) calling journey is a case in point:

*I was a student at the University X. On 14 February, I was arrested and went to jail. When I was there, I dreamt of a cross… After six months, I was released from prison. I went back home and began to work at Company X. I decided to complete school… I decided to take the ministry, in this course of Theology. [Before my arrest] at the time, I was young, at the age of 11 or 12, I dreamt that my grandfather came to me and gave me a stick and a bible.*

Calling for participants in FG2 provides them with the necessary emotional and spiritual well-being and resilience in the face of hardships, as stated by Werner (FG2):

*The divine calling is not only a calling to be a pastor, it’s a calling to be a disciple first and take up your cross and carry it. So many times, it is the one thing that carries me because you recognize [the challenge] of carrying the cross … to avoid being self-indulgent … you chose it the day that you said yes to God.*

Ultimately, whatever initiated the sense of calling, it is the internal calling and one’s relationship with God that are emphasized in fostering spiritual well-being. Neels (FG2) notes: “*A congregation calls you. Sometimes you get hurt in your experience of the external calling… it is not the same as your internal calling from God”*. Herman (FG2) explains his internal calling as a personal connection with God, giving him spiritual resilience to serve: “*It is the faith or the grace of God that endures in you, that is the core. So, it’s a subjective thing … we believe it’s God’s grace that holds you, that in spite of what you experience, or how the congregation cares for you or not. The body is wounded, but we are still a family and should be spiritual leaders by grace”.*

Calling was thus described as a uniquely personal spiritual journey that ensued because of life experiences and interpersonal relationships, leading to a deep connection with God. The participants emphasized that calling is an emotional journey because it requires personal sacrifices in serving others (for example, congregation, community, family/friends [FG1, FG2, and FG3]). This lead to labelling the theme an altruistic calling, because it is a calling to serve God by serving others. The participants explained that their calling begins with God as their service is to purposefully be servants of Jesus Christ, also known as being disciples of Jesus.

#### Discipleship

Discipleship was constructed from the affinities of servant leadership (primary driver, FG3), family building (pivot, FG3), spirituality (secondary driver, FG1), caring (primary outcome, FG1), and caregiving (secondary outcome, FG2). As it follows logically from the participants’ sense of calling, we ultimately constructed it as a primary outcome in understanding spiritual well-being.

In FG3, servant leadership as affinity was described as responding to the participants’ calling from God. Being a servant leader involves gratitude for being called and results in an obligation to spread the Word of God and bring hope to individuals, groups, organizations and communities: “*To be a servant and a true servant allows one to accept the calling, I am called … [to] proclaiming the Word is not the only goal but also doing things in a practical way, visiting the sick”* (Johannes, FG 3). In FG3, family building as an affinity referred to how the pastors grow in their spiritual maturity due to the challenges; they face in being part of a large congregational family and how these challenges offer personal growth and strength opportunities. To illustrate, Mathew (FG3) refers to personal sacrifice as the key to understanding the extent and nature of discipleship: “*As pastors we should give support during bereavement… we need to be there when people are in need … You cannot choose your family if someone in the congregation passed away. You must be there. You are the face of the church”*. Matthew’s words demonstrate how family building extends beyond one’s own family, emphasizing that serving others is priority of discipleship because one becomes an image-bearer of the Church.

The affinity called spirituality emerged in FG1, yet the meaning ascribed to it is similar to servant leadership and family building, as participants linked spirituality to being missionaries of God, sent by God to spread his Gospel to others: “*Primary to my spiritual well-being is my relationship with God … I experience the same as a missionary of God”* (Bernard, FG1). To be a missionary or a servant of God (i.e., discipleship), FG1 participants emphasized the need to constantly engage in spiritual practices to nurture and strengthen their well-being because working with congregations and other people involves traumatic experiences. It can be hurtful and emotionally draining. It is through their spirituality that they receive strength to be disciples and guide others’ healing through what they described as the affinity, caring: “*I would like to describe healing that in my pain my relationship with God is not broken. Even in my pain, my hurt and disappointment, it is not a hopeless situation”* (Jaco, FG1) and to be a disciple it “*is important that you become quiet … to spend time with the Word through prayer”* (Tessa, FG1).

Like caring (FG1), care giving (FG2), wherein participants describe service to others as caregiving in pursuit of spreading God’s love and grace, was included in synthesizing the meaning of discipleship. In the words of Herman (FG2), caregiving entails “*Meaningful engagement; it’s about making a difference and becoming involved in the predicament, the hurt, the needs, or to be human to others”*. Caregiving is described likewise by Marco (FG2): “*Compassion, care, love. Observing and being sensitive to the emotions of others”*, and consequently linked to spiritual practice: *“I am stunned by the wonder of prayer and God and the reality of the Holy Ghost”* to bring *“comfort and nurturing of people”*. In FG2, Peet reflected on the difficulty of caring, emphasizing the need for spiritual practices in discipleship:

*It is as if one does not “care” enough, because in death you stand with someone, but then there is another one that passes on. Then you have this guilt that you did not attend to a person. It all happens so quickly. Then you have to get to a sick person, and then you are with someone who has lost a loved one. And then you lie in bed at night and think, oh, I have not done this yet.*

Discipleship is understood to represent the serving of others because of the conviction to serve God and spread His gospel. It entails personal sacrifice and hardship and therefore requires a strong spiritual practice. The participants reflected that the purpose of serving others does not include a strategic organizational business plan but rather a divine calling from God. Discipleship thus follows calling.

#### Seasons of the ministry

To construct a meaningful understanding of the theme *seasons of the ministry*, six affinities were conceptually integrated, namely, *seasons of the ministry* and *self-development* (pivots, FG1), *developmental influences* (primary driver, FG2), *crisis* and *conflict management* (primary outcomes for FG2 & FG3), and *career journey* (pivot, FG3). On average, seasons of the ministry presented as a circular/pivot.

In FG1, the evolving nature of spiritual well-being was summarized aptly by Jaco (FG1) as that “*phases in the ministry*” relates to “*phases in well-being*”. The affinity, self-development was constructed by FG1 as an ongoing process of self-discovery and deep learning about the self in relation to God and others, as evident in the words of Peet (FG1): “*and at times I was lonely … I constantly had to prove myself and it brought me to a point which has led to burnout … The other congregations in which I was employed with, form part of my development*”. Self-development as core to building spiritual well-being is noted in Diederick’s (FG1) reflection on moving through the phases of his ministry and shows how his spiritual well-being developed in terms of growing self-awareness and knowledge:

*It is a new beginning for your calling, to say that you move to a new stage of in your ministry … Throughout my life I’ve experienced various times and incidents that helped me towards learning new things and learning about myself … During my first recognition of my calling, I experienced these up-and-down emotions about my personal and professional journey. Good times and bad times. It is typical on those points, despite the strong calling of your work as a pastor, to ask whether you are serious with the things that you are busy with. Embrace the good and the bad times, moments that help you to grow and develop to be the person that God wants you to be and to live.*

In the same manner, Francois (FG1) reflected on growing spiritual resilience: “*It is about your personal and professional journey to become a pastor. My journey was difficult and lengthy. It is almost the difficult part that is behind you and it is the difficult situations that God put on your journey to use. I almost want to say it is the hurt of the past and the difficult things that God use to make you stronger”.*

Crises and conflict management situations were discussed in FG2 and referred to as developmental influences forming and strengthening their spiritual resilience. Herman (FG2) reflected: “*If there is a crisis in a congregation, the pastor, no matter whether it is right or wrong, is always in the line of fire and some will agree with how you manage the crisis, and some will crucify you. You cannot really win. You are drawn into something that you do not like or even trying not to be involved with. Crisis in the church cause crisis in the ministry, crisis in relationships, and crises in your own life*. Again, the crises and conflicts initiate a continuous process of self-questioning and self-reflection in relation to God and others. This is demonstrated in how Werner (FG2) reflects on the self-imposed and other-imposed expectations that challenged his sense of self in relation to God. He says the “*feeling that I’m supposed to be a professional religious person without faults”* caused self-doubt and continues to explain how as his spiritual maturity grew, he coped better: “*I had to differentiate whether the ministry is only a religious occupation or is the ministry a passion that I believe the Lord has called me”*. As such, participants in FG2 related their evolving spiritual maturity and resilience to how they grow in their calling (relationship with God) and discipleship (serving others) through self-development.

Referring to the initial stage of his career Johannes (FG3) reflects on how his relationship with God evolved and gave him inner strength during the first season of his ministry: “*When I was called is when I realized I am confident*”. It seems that the challenges they face in the different phases of their ministry, challenging them with crises and conflict management, leads to building spiritual maturity and resilience. In this regard, Moses (FG3) notes how starting his studies was much easier as opposed to the spiritual challenges he experienced when he started to work in the congregation: “*When I was at school, I was the best student but when I did my practical it was so different. To see how difficult life and the congregants are”*. The sense that spiritual well-being is strengthened through continuous self-reflection concerning one’s calling and discipleship was also emphasized in FG3 by Moses when he spoke about how he changed spiritually in his career journey: “*As part of my personality and my calling, I reflected on my spirituality and really think deeper about being a pastor in the world. It really changed my life*”.

FG1 initially coined and constructed the affinity *seasons in the ministry*, which became the label for the synthesized theme. Seasons of the ministry related to how pastors’ spiritual well-being is reflected in a level of spiritual maturity and resilience that evolves throughout their ministry through a process of consistent self-reflection and deep learning about the self in relation to God and others. The pastor’s developmental phases and work-life experiences lead to self-discovery and growth always in the context of their calling and discipleship.

#### Ethics

Ethics was constructed from the affinities labelled *occupational authenticity* (pivot, FG2) and *success* (pivot, FG3) as well as *own value* (secondary outcome, FG1). Overall, from the narrative synthesis, we derived it from functioning as a pivot because it describes how the primary driver, c*alling*, and the primary outcome, *discipleship*, is taken up.

Various ethical dilemmas in the ministry were extensively highlighted across the focus groups as taxing the pastors’ integrity, ethical conduct, and well-being. Some included narratives of incidents involving fraud and theft of congregation funds, accusations of sexual harassment, expressed disappointments and negative feedback, and gender discrimination. In response to their calling and discipleship, pastors feel they must demonstrate ethics in the face of temptations and challenges. Their ethics is thus rooted in a sense of responsibility to live their altruistic calling and discipleship, irrespective of the nature of the service or sacrifice required and often resulting in criticism, rejection or personal attacks. They were able to demonstrate ethical conduct or ethics as central to evaluating their own spiritual well-being. Ethics are described by reflecting on their occupational authenticity, success experiences and by valuing themselves.

Occupational authenticity was constructed in FG2 and described as remaining faithful to their calling and discipleship in the face of the lonely and often negatively judged pastor role. The loneliness of the pastor role is emphasized by Herman (FG2), but his relationship with God strengthens him to stay true to the occupation: “*You are quite burdened, you make decisions abundantly, and you do not talk to anybody about it except with God*”. Herman (FG2) continues to describe the strength that is found through occupational authenticity: “*To me that loneliness is not just a negative thing it is sometimes a good thing”*. Participants in FG1 shared the notion of the lonely role of the pastor encumbered with unique, anomalous, sometimes divine, or super-human expectations: “*There are only a few professions where you are the paid official, actually the employee, but also in a way the employer; you set the pace but contribute like everybody else to the congregation”* (Marco, FG1). For FG1, their ability to remain faithful to their calling and discipleship in this context, is strengthened through spiritual values and beliefs as reflected in the affinity called *own value*. As a female pastor, Tessa (FG1) shared painful experiences of prejudices and stereotypes from her congregation. She reflects on her ethical values (humility and transcendence) that help her to respond to these experiences: “*To stand up for my belief with humbleness and for what God wants to do through me as the focus should be on God not on you”*. For Jaco (FG1) own value means living according to one’s spiritual values and leads to valuing himself (being authentic to the self): “*Since I am in the ministry, there were a few things that I told myself from the beginning, to be spontaneous, transparent and to be me. I am who I am. If you see me and hear me, then you know me. This adds to my well-being. I do not need to pretend*”.

According to FG3, the affinity *success* means that as a pastor you were able to conduct yourself with respect and build positive relations among colleagues and congregational members. FG3 participants emphasized nurturing moral principles and values as essential to building relationships: “*We need to have moral discipline* … *morality is important, respecting other people’s culture”* (Johannes, FG3). To emphasize spiritual values as key to the success of the pastor, Mathew (FG3) notes the moral conduct of the pastor as distinctive from other people: *“The church is the salt and makes a difference in other people’s lives. Even though you mingle with them you do not do what they do”*. Ethics conceptualize the manner in which pastors have to conduct themselves to preserve their sense of self, the relationship with God (their calling) and their commitment to service (discipleship). Neels (FG2) emphasized wise decisions and actions in the context of the pastor being an occupation that is vulnerable to ethical scrutiny: “*Thus, pastors need to take precautions so that they do not find themselves in situations where they may be vulnerable to such charges*. *We must act with great wisdom at all times”.*

All three focus groups reported that their spiritual well-being is impacted by a unique ethically challenging occupational context that requires them to act professionally and ethically. Ethical conduct as a pastor, however, moves beyond upholding right and wrong. It is inspired by a deeply internalized perspective of the self in relation to God and other people. Ultimately, their ethics becomes a measure for participants to determine whether they are functioning well at work. When they lose their calm, act unwisely, and from uncontained emotion, their spiritual well-being falters.

## Discussion

The aim of this paper was to describe pastors’ spiritual well-being as a fundamental part of their overall well-being or flourishing. Four themes were constructed to describe pastors’ spiritual well-being. Altruistic calling and discipleship emerged as the primary pillars of pastors’ spiritual well-being. Seasons of the ministry demonstrate the evolving nature of their spiritual well-being and ethics, represent how they enact their pastor role daily.

Calling was a predominant narrative in all affinity constructions in all the focus groups, corroborating Bledsoe and Setterlund’s (2015) proposition that calling is a non-negotiable in pastors’ ability to thrive and flourish in their occupation. Calling has become an essential notion in career well-being (Lysova et al., 2019). In the work context, it is also referred to as a vocation, yet it remains based on engaging in meaningful and purposeful tasks and in being motivated by other-directed values (Dik & Duffy, 2009). The idea of calling as a “transcendent summons” (Dik & Duffy, 2009, p. 427) corresponds with the existential dimension central to understanding spiritual well-being (Malinakova et al., 2019). In this study, calling is regarded as central to pastors’ spiritual well-being and is specifically noted to be an altruistic calling. Psychology literature distinguishes two forms of calling, namely self-orientated calling (for self-fulfilment) and other-orientated (calling for serving others) (Michaelson & Tosti-Kharas, 2019). In the context of this study, calling was notably other-directed which is congruent to how calling is typically regarded as a communal phenomenon in the Christian church context (Nel & Scholtz, 2015) and as a summons from a higher power external to the self (Dik & Duffy, 2009). Participants’ altruistic calling enabled a deep connection with God and directed them to choose a vocation in the ministry that requires personal sacrifices in servicing others. Altruistic calling also aligns with J. Fisher’s (2010) explanation of self-awareness as a transcendent drive that manifests in the personal domain of spiritual well-being. Through self-awareness participants became more attuned to their altruistic calling because it offers them meaning and purpose. Their calling is self-transcendent because it encapsulates the opportunity to cure or care for human souls (*cura animarum)* (Louw, 2017) and as such an altruistic calling manifests in way of living every day that leads to discipleship.

As discipleship followed logically from the participants’ altruistic calling, we constructed it as a primary outcome in understanding spiritual well-being and thus regard it, together with calling as the primary pillars describing the vertical dimension (see Alorani & Alradaydeh, 2018) of the pastor’s spiritual well-being. Discipleship is the participants’ response to God’s calling to serve Him by spreading His gospel and caring for the congregation. In Christian literature discipleship is primarily described as a response to the biblical call to love God above all, to deny and fully surrender the self and abandon all one’s worldly possessions to follow God (see Luke 14:26–33; Luke 9: 24–25; Matt 16:24). It is to become *image-bearers* of God’s goodness to care for those in need (Anderson & Skinner, 2019; Nel, 2017). Similarly, Niemandt (2016) describes discipleship as a positive response to God’s calling to engage in His mission, which entails a life orientation of generous service and care for the weak. The communal domain of spiritual well-being (J. Fisher, 2010) and the inherent relational nature of discipleship (Himes, 2011) reflect discipleship as manifested in this study, because it denotes the interpersonal love and care that is expressed by pastors when they speak about being a disciple. Discipleship is communal practice (Himes, 2011) which relates to how in this study, discipleship describes participants’ everyday role of service to others, which requires personal sacrifice and endurance through the emotional and physical hardships of daily life. This notion of personal sacrifice is a longstanding characteristic of discipleship from a Christian perspective (see Drissi, 2019; Onyinah, 2017). Discipleship entails servanthood at the cost of self and requires one to be willing to deny the self (Anderson & Skinner, 2019). The notion of transformative discipleship in recent literature has relevance as it points to how responding to God’s call changes the way you live your life (Drissi, 2019) and ultimately changes your identity (Anderson & Skinner, 2019) to become like Jesus Christ (Onyinah, 2017). Participants of this study engage in daily spiritual practices for the inner strength and self-sacrifice needed to be a disciple. Their engagement with activities to grow spiritually relates to discipleship practices of prayer, meditation, fellowship and studying the bible (Drissi, 2019; Himes, 2011) and are required in different ways and to different extents depending on the seasons of the ministry in which the pastors find themselves.

With reference to the third theme, the seasons of the ministry reflect pastors’ continuous development and the growing spiritual maturity for they experience in their different career and life stages. Participants’ spiritual well-being is dependent on a process of deep learning that ensues and evolves with continuous self-reflection to make sense of their altruistic calling. Seasons of the ministry centre on a growing spiritual maturity and resilience through consistent reflection on the self in relation to God and others. The sense of spiritual maturity and spiritual resilience reflects the experience of spiritual well-being. Throughout the seasons of ministry, pastors’ calling is *strengthened* and *strengthens* their identity as a disciple of God, thus growing their spiritual well-being in an evolving and dynamic manner. Nzonzo (2017) similarly views spirituality in an organizational context as an ongoing process of pursuing meaning and purpose and consistently enhancing the quality of relationships. Dhar et al. (2013, p. 3) speak of spiritual health in the same evolving, dynamic way, as a process of “becoming” unfolding into “being”; a self-evolution process in pursuit of realizing one’s potential and living a life with meaning and purpose. This evolving spiritual maturity reflects how growing self-awareness leads to establishing identity in J. W. Fisher et al. (2000, 2010) personal domain of spiritual well-being and the self-integration and intrapersonal strength resources in the horizontal dimension of spiritual well-being (Malinakova et al., 2019). Spiritual well-being develops through constant self-exploration and deep learning, similar to introspection and reflection highlighted by Dhar et al. (2013) as key tools in an evolving spirituality. This intra-personal process of introspection in relation to pastors’ calling and discipleship seems akin to the phenomenon of identity work. Identity work refers to the continuous process of identity construction and self-verification that result from one’s need for a unique sense of self and one’s need to belong (Stets & Serpe, 2013; Sveningsson & Alvesson, 2003). The pastors’ identity work revolves around their altruistic calling and discipleship and fits well into the personal domain of J. W. Fisher’s et al. (2000) spiritual well-being framework, which deals with how one intra-relates with the self about your meaning and purpose (Gomez & Fisher, 2005). The seasons of ministry describe how spiritual well-being continues to change and evolve through self-reflective identity work throughout pastors’ career-life journey.

Ethics applies to pastors’ spiritual well-being as it represents the extent to which their behaviour stems from an authentic and value-driven orientation to act in accordance with the self in the context of their calling and discipleship. In the IQA results, we noted that ethics becomes a way to measure whether they function well at work, always within the context of their altruistic calling and discipleship. Their altruistic calling and discipleship represent the unique standards of being against which all behavioural responses are evaluated. As such, ethical conduct is reflected in responding to difficult situations and people calmly, with wisdom and selfless self-regard. Van Dierendonck

Dierendonck (2004) similarly links spiritual well-being to the ability to access inner resources and living authentically according to one’s inner truth. When pastors in this study managed to act ethically, it strengthens confidence in their inner spiritual resources and thus strengthens their spiritual well-being (see Van Dierendonck, 2004). Ethics is a driving force for pastors and reflects a standard of being and doing. Through reflective self-awareness pastors continuously assess how they need to cure or care for the human soul (Louw, 2017) in being authentic and transparent on an intra- and interpersonal level and constantly ask whether they live their values in life. Hence, ethics in this study demonstrates ethical professionalism or conduct that is reflective of the beliefs fundamental to their calling and discipleship (Rudolph, 2019).

From the above discussion, a dynamic interrelatedness is evident between the four themes. Using J. Fisher’s (2010) spiritual well-being framework as a guide allows for an integrated conceptual description of pastors’ spiritual well-being. The pillars of spiritual well-being, an altruistic calling and discipleship, seem to respectively correspond to the transcendental and communal well-being domains identified in J. Fisher’s (2010) spiritual health framework. The transcendental domain concerns the relationship between the self with a transcendent or divine reality (J. Fisher, 2010). The altruistic calling constructed in this study is similarly related to the pastors’ relationship with God, resulting in a transcendent response to dedicate their lives to God and others. A sense of calling provides people with purpose and meaning and leads to commitment and dedication to a cause (Yang & Fry, 2018) of serving others (Louw, 2017, 2018). So, do the pastors in this study respond to their calling by becoming servants of God’s people, resulting in discipleship? For pastors, discipleship is about serving and caring for others to build God’s family. These two pillars are further mirrored in how models of spiritual leadership include calling and membership as the key components to leaders’ spiritual well-being (Egel & Fry, 2017; Gotsis & Grimani, 2017; Yang & Fry, 2018). In most definitions of spiritual well-being, establishing a connection with a higher power and with others are emphasized (Gomez & Fisher, 2005). For the pastors in this study, it seems that these self-other relationships lie in their unique experience of being called and responding to this call with service (discipleship), resulting in an affirmation of a meaningful and other-connected self-identity. Spiritual well-being is thus experienced when the pastor experiences existential meaning and purpose through their discipleship and in congruence with their altruistic calling.

## Conclusion

Positive psychology emphasizes how consistent efforts to enhance employees’ well-being is essential to optimal functioning at work (Arya et al., 2017). In the field of positive psychology, flourishing is a comprehensive, multidimensional well-being model for application in the workplace (Janse van Rensburg et al., 2017; Rothmann, 2013). Despite the notion that well-being is contingent on a sense of wholeness and integrating all dimensions of the self, including the spiritual (Gomez & Fisher, 2005), the flourishing framework excludes it as an explicit and primary sub-dimension of employee well-being. While conceptualizations of flourishing equate it to a state of complete human well-being, which is inclusive of constructs such as meaning and purpose (VanderWeele, 2017), the flourishing framework only highlights meaning and purpose as elements of psychological well-being. Scholars have called for spiritual well-being to be incorporated into the overall concept of well-being (Dhar et al., 2013; Van Dierendonck, 2004; Ziapour et al., 2017). From this study, we learned that in the Christian context pastors’ well-being extends beyond the elements of meaning and purpose that underlie the psychological well-being dimension in the flourishing framework (see Rothmann, 2013). Meta-theoretically, the present study extends the flourishing framework by proposing spiritual well-being as an additional yet explicit and primary well-being sub-dimension for Christian pastors. This however also speaks to the limitation of a qualitative context-specific study such as the current one. The findings may be transferred to similar Christian contexts relevant to South Africa, however, we cannot claim any generalization in our proposal to incorporate spiritual well-being as conceptualized here to the flourishing of employees in different contexts.

In conclusion, the findings demonstrate that to flourish in the work context of the ministry, spiritual well-being is essential and should be highlighted as such. In this respect and through this study, we have provided a data-driven and meta-theoretical rationale for spiritual well-being to position it as a key dimension and determinant of flourishing in the Christian ministry. More research is needed on how other employees would benefit from spiritual well-being. To maintain pastors’ well-being in their challenging work context and role, specific attention should be given to facilitating processes of self-reflective to secure their sense of altruistic calling and discipleship throughout the seasons of their ministry. The scope for studying the spiritual well-being of pastors in different religious and societal contexts thus remains imperative.

## Notes

1. The **Cinderella metaphor** is about unrecognized value—someone useful and important yet whose importance has for some time been ignored or neglected (Deacon, 1993; Esterhuysen, 2012).

2. The first author was the facilitator and primary researcher of the study.
